# Augmented authenticity in athlete branding through human-AI co-production

**DOI:** 10.3389/fspor.2025.1643885

**Published:** 2025-10-02

**Authors:** Hans Westerbeek

**Affiliations:** ^1^ Institute for Sustainable Industries & Liveable Cities (ISILC), Victoria University, Melbourne, VIC, Australia; ^2^ Victoria University Business School, Melbourne, VIC, Australia

**Keywords:** athlete branding, human-AI co-production, sport marketing, influencer culture, AI ethics, digital platforms, human-led branding with AI support, algorithmic co-production

## Abstract

In the age of generative AI and digital self-production, athlete branding is undergoing a profound transformation. This conceptual paper introduces the idea of augmented authenticity to describe the co-creation of athlete identity through both human narrative and machine-generated content. Athletes today can operate as digital producers and autonomous entrepreneurs who increasingly rely on AI to scale engagement, personalise content, and deepen fan relationships. Drawing on literature from sport marketing, influencer culture, AI ethics, and digital platforms, this paper develops a scenario-based framework to explore how augmented authenticity may evolve. Three future scenarios, human-led branding with AI support, algorithmic co-production, and synthetic substitution, are used to stress-test strategic, ethical, and governance implications. The paper highlights how generative AI can empower underrepresented athletes while also creating new risks of losing control over the narrative and displacement of the athlete's identity. It argues that current sport governance models are ill-equipped for these shifts and proposes athlete-centred regulatory strategies to protect the control that athletes have over the narrative sovereignty, and their psychological well-being. Ultimately, the paper invites scholars, practitioners, and policymakers to engage with the future of athlete representation not merely as a technical challenge but as a philosophical question of what it means to be human in the platform age.

## Introduction

What does it mean to be human in an age when machines can speak for us, represent us, and even feel, at least convincingly, on our behalf? In his sweeping reflections on the trajectory of human civilisation, Harari (1, 2) suggests that the defining trait of Homo sapiens has always been our capacity to create and believe (and trust) in shared fictions that become our reality, from religions to nations to brands. As we enter the age of artificial intelligence, Harari warns that humans may surrender the authorship of these fictions to non-conscious but hyper-capable algorithms. In doing so, we risk losing our monopoly on meaning-making. Harari (1) argues that as authority shifts from humans to algorithms, we may reach a point where we no longer know ourselves better than algorithms know us. It can be argued that nowhere is this shift more visible than in the realm of sport. Athletes, once symbols of raw human excellence and embodied authenticity, are increasingly surrounded by layers of digital mediation. From AI-curated social media feeds to voice-cloned sponsorship messages and synthetic training simulations, the modern athlete operates in a space where the boundary between person and persona is algorithmically blurred. As Harari might put it, the athlete is no longer just a body in motion or a voice in the arena, but a data node in a system of narrative computation. This article introduces the concept of augmented authenticity to make sense of this evolving condition. It describes a new logic of athlete branding in which identity is co-constructed by human intention and generative AI. In this paradigm, athletes engage fans not through unmediated “realness” alone, but through hybrid expressions, deeply personal yet digitally optimised, emotionally resonant yet partially synthetic. From AI-personalised messages and generative highlight reels to fully digital avatars, branding is no longer merely communicated, it is calculated. While the cultural and commercial impact of generative AI is rapidly growing, the implications for athlete branding, and the integrity of athlete-fan relationships remain under-theorised. This paper synthesises recent scholarship in sport marketing, influencer culture, AI ethics, and digital governance to conceptualise augmented authenticity as a reflection of the rapidly changing space of sport fandom. Through this lens, I argue that authenticity is no longer a binary of real or fake, but a spectrum, fluid, strategic, and increasingly co-authored by machines. Augmented authenticity as a concept therefore is treated as a continuum of human-machine co-production. This continuum spans from human-led authenticity, through algorithmic co-production, to synthetic substitution, reflecting the shifting degrees of athlete agency and machine authorship in branding. The paper proceeds in five parts. First, foundational sport marketing theory is revisited to situate authenticity as a core value proposition in athlete branding. Next, it is explored how generative technologies disrupt traditional branding logics, prompting a redefinition of trust and narrative ownership. A scenario-based framework to imagine how augmented authenticity may evolve is then proposed. Finally, governance and ethical implications of synthetic identity in sport are discussed followed by a research agenda for future inquiry. Overall, this paper seeks to confirm that the interplay between humans and machines to curate stories is a technological development but it is also a civilisational crossing in the road. Will the augmented athlete become a more empowered storyteller, or a commodified construct optimised for engagement metrics? The answer will shape not only the future of athlete branding, but the meaning of the role of humans in sport.

## Theoretical background

Authenticity has long stood as a key concept in sport marketing theory. Early scholarship emphasised the unique emotional bond between athletes and fans, a bond rooted in perceived connection and shared understanding of the human story (3, 4). Unlike corporate brands, athlete brands were thought to derive value not merely from performance, but from the alignment of public persona with private self. The congruence between an athlete's value and actions formed the basis of a trustful rapport and as an outcome, a commercially viable relationship. Drawing on parasocial interaction theory (5), scholars such as Doyle et al. (6) and Su et al. (7) demonstrated how fans develop emotionally intimate, one-sided relationships with athletes. These relationships are sustained not just by visibility, but by authenticity: the sense that the athlete is accessible, honest, and “real.” In the branding literature, this has been reinforced by constructs such as self-brand congruity and personal brand identity, which position authenticity as a communicative strategy and a relational currency (8, 9). However, this framework presumes human authorship and narrative control. It assumes that athletes, or their teams, craft the stories that fans consume. This assumption becomes increasingly unstable in the context of generative AI, where brand communication is not just mediated by platforms but co-produced by autonomous systems. In this landscape, athlete content may be algorithmically drafted, stylistically cloned, or fully synthesised without direct human involvement. As Harari (1) warns, when algorithms know us better than we know ourselves, the line between authorship and automation dissolves. The influencer marketing literature provides a useful bridge here, introducing the concept of constructed authenticity, the idea that “being real” is itself a form of strategic performance shaped by platform logics and audience feedback (10, 11). On platforms like Instagram and TikTok, authenticity is not an essential trait, but a malleable brand attribute, co-created through a dynamic loop of performance, response, and algorithmic curation. This performativity becomes even more potent, and potentially more problematic, in sport contexts, where narratives of resilience, and identity often underpin fan allegiance. The term “performativity” originates in linguistic philosophy and gender theory, for example in the work of Butler (12), who argued that identity is not a fixed essence but constituted through repeated acts that acquire social meaning over time. In the context of digital sport branding, performativity refers to how authenticity is strategically enacted, often in response to platform algorithms and audience expectations, rather than naturally expressed. When these narratives are enhanced or even fabricated by AI systems, traditional notions of trust, integrity, and brand legitimacy are strained. As Gao et al. (13) and Xu and Baghaei (14) note in their work on AI ethics, the proliferation of synthetic content, from deepfakes to digital clones, poses new risks to identity integrity and audience deception. It is within this rapidly evolving terrain that I introduce the concept of “augmented authenticity”. Operationally, augmented authenticity can be understood as a continuum rather than a fixed state. At one end lies human-led authenticity, where athletes remain the primary authors of their stories and use AI only as a supporting tool. In the middle lies algorithmic co-production, in which athletes and AI systems share authorship, and authenticity is experienced as a hybrid outcome of human input and algorithmic optimisation. At the far end lies synthetic substitution, where the athlete's identity is simulated by digital proxies with little or no direct involvement. This framing positions augmented authenticity as the varying degrees of human–machine authorship in athlete branding, and as a dynamic condition that fans evaluate in terms of trust, realism, and emotional resonance. This is not merely a new aesthetic of branding, it is a new epistemology (how do we know it is “real”?) of athlete identity, in which human stories are told with, rather than by, machines. It recognises that athletes remain central to their brands but increasingly share narrative authorship with generative systems. Augmented authenticity, then, is the product of hybrid storytelling, part lived experience, part machine-optimised projection, filtered through algorithmic metrics of engagement and relatability. In sport marketing terms, this marks a shift from managing image to managing ontology (what is “real”). The marketing focus expands from not only, how athletes look, but how they are constructed and known in a platformed, programmable public sphere. As augmented authenticity in the AI-driven platform economy is a new lens, I am conducting a conceptual analysis and development process that is driven by scenario planning.

## Methodological approach: conceptual analysis and scenario planning

This paper adopts a conceptual analysis methodology aimed at theorising the emerging construct of augmented authenticity in athlete branding. Conceptual research, as defined by MacInnis (15), serves to clarify, extend, or create theoretical constructs that are underdeveloped or not yet empirically captured. In this case, augmented authenticity refers to the hybrid condition wherein athlete identity and brand communications are co-produced by both human agency and generative artificial intelligence. As the technological landscape evolves faster than academic literature can empirically verify, conceptual work is essential to offer anticipatory frameworks, guide future research, and inform practical governance responses. To operationalise this conceptual inquiry, the paper employs a scenario planning approach (16). Scenario planning is not a predictive method but a structured process of imagining multiple plausible futures under conditions of uncertainty and disruption. It is particularly suited to contexts, like generative AI in sport, where technological, ethical, and socio-cultural trajectories are volatile and intertwined. Scenarios serve as “stress tests” for new concepts, helping scholars and practitioners evaluate their relevance and where boundaries might be across divergent futures. Here, scenario planning is applied to map three illustrative futures of athlete branding, each reflecting a different degree of human vs. algorithmic authorship. First, I will consider human-led authenticity with AI support. This is where the athlete retains narrative control and uses AI as a tool to scale or streamline content production. I then move on to algorithmic co-production. This is where AI systems significantly shape content tone, scheduling, and fan interaction, with limited athlete oversight. The final scenario considers the perspective of synthetic substitution. This is where the athlete's identity is fully simulated via digital avatars, deepfakes, or voice clones, raising foundational questions about representation, who is the author of the story, who tells the story and who approves of it. These scenarios are analytically derived from a synthesis of current peer-reviewed literature in sport marketing, influencer branding, generative AI, digital ethics, and human–machine interaction. They are not fictional thought experiments, but structured extrapolations from observable trends and documented use cases (e.g., 11, 14, 17). Each scenario is used to probe the theoretical robustness of augmented authenticity, explore its relational and ethical dimensions, and identify implications for athlete-fan connection, brand governance, and digital labour. The methodological design also aligns with Harari's (1, 2) philosophical provocation, that as humans begin to outsource identity construction to algorithms, our social and institutional frameworks must adapt. Scenario planning, in this context, becomes not only a strategic tool but a philosophical exercise. I use it to rehearse the moral and managerial choices that lie ahead in the algorithmically mediated sport economy. While scenario planning offers a structured way to stress-test emerging concepts, its use here also introduces limitations. The analysis is conceptual rather than empirical, and the scenarios are illustrative extrapolations from current literature and trends rather than predictive models.

## Future scenarios of augmented authenticity

Across three scenarios, athlete agency is treated as a variable rather than a fixed state. Agency is strong where AI plays a minor supportive role, partial when authorship is shared and minimal when athlete identity is substituted by machines. To examine how augmented authenticity may evolve in the rapidly transforming landscape of athlete branding, this section therefore introduces three analytically derived scenarios. Each scenario represents a distinct configuration of human and algorithmic agency in identity construction and brand communication. The scenarios explore possible trajectories based on existing literature, technological trends, and shifting athlete-fan dynamics. By reflecting on the concept of augmented authenticity across divergent futures, the scenarios illuminate its strategic challenges but also ethical contours. Informed by Harari's (1) thesis that the authority to shape narratives is being transferred from humans to algorithms, these scenarios invite us to interrogate what happens when being human, in branding terms, no longer requires being present. They also build on foundational sport marketing principles that position authenticity, trust, and narrative congruence as core to the emotional connection of athlete-fan relations (4, 6).

### Scenario 1: human-led authenticity with AI support

In this future, generative AI functions as a support system, not a substitute. Athletes retain creative control over their personal brand and deploy AI tools selectively, for content scheduling, language translation, image enhancement, or highlight editing. Branding remains rooted in lived experience, personal values, and direct authorship, with AI serving to optimise reach and consistency. This scenario aligns with current branding practices where authenticity is preserved through close alignment between athlete identity and audience perception. As Brison and Geurin (18) and Nichols and Shapiro (11) note, fan trust remains strongest when athletes are perceived as emotionally accessible and narratively coherent. The co-creation of meaning between athlete and audience, emphasised by Anderski et al. (19), is facilitated, not displaced, by technology. Here, augmented authenticity is incremental. Athletes extend their voice through AI without relinquishing narrative authority. The risk of audience alienation is minimal, and the athlete-fan relationship deepens through AI-enhanced intimacy.

### Scenario 2: algorithmic co-production

This intermediate future envisions a shared authorship model, where AI systems are embedded not only in the distribution but also in the production of brand content. Generative algorithms draft captions, simulate tone, respond to fans, and optimise timing based on audience engagement data. Athletes provide input but play a secondary, editorial role. This reflects Bleier et al.'s (17) account of the creator economy, where content is co-authored by people and platforms, and algorithmic logic increasingly determines what is seen, liked, and monetised. Gao et al. (13) and Xu and Baghaei (14) similarly identify the growth of predictive AI tools that shape both message and moment. In this scenario, augmented authenticity becomes strategically synthetic. “Realness” is approximated through machine learning models trained on the athlete's digital history. While this can increase engagement, it also destabilises traditional trust signals. Fans may enjoy the content but question whether the athlete is “really there,” leading to a paradox of emotional proximity without relational certainty, or “I feel closer to my hero… but am I?” The ethical tension intensifies. Is the athlete responsible for an AI-generated misstep? Does the audience have the right to know who, or what, authored the message? The brand becomes more efficient, but the boundary between person and persona becomes more porous.

### Scenario 3: synthetic substitution

This high-disruption future imagines the athlete brand as a fully synthetic entity, an avatar, voice clone, or AI-generated persona that interacts with fans, sells products, and even gives interviews without direct athlete involvement. The athlete licenses their likeness to an agency or platform, which deploys it across time zones and campaigns in perpetuity. Momenifar et al. (20) and Carrio Sampedro (21) highlight how such substitution models are already emerging, driven by commercial logic and content scalability. Li and Huang (22) show that AI-generated brand figures are being used in parallel industries to maintain constant audience engagement without human fatigue. In this scenario, augmented authenticity gives way to synthetic credibility. The illusion of presence and personality without the presence of the person. Fans may still feel emotionally connected, especially as deepfakes and avatars become indistinguishable from human output. However, the relationship becomes ontologically ambiguous, real in feeling, unreal in source. This scenario raises profound questions about identity ownership, emotional manipulation, and the limits of digital embodiment. It is here that Harari's warning becomes most tangible in that the athlete is no longer the author of their own story but the licensor of their narrative shell. What remains authentic may not be the athlete, but the algorithm's capacity to simulate them believably. As such, synthetic substitution presents a paradox. It can extend athlete presence and commercial opportunity, even as it risks undermining narrative sovereignty and eroding the very authenticity on which athlete-fan connections are built.

## Strategic and ethical implications

The integration of generative AI into athlete branding brings with it a profound shift in how athlete identity is constructed and how this leads to a valuable fan-athlete relationship that is built on trust. Augmented authenticity is not merely a tactical innovation, it is a reconfiguration of who tells and owns the story and how this translates to fan relationships. It creates a human-machine boundary that defines what it means to be an athlete in public life. As Harari (1, 2) warns, when storytelling power migrates from individuals to algorithms, the authority to shape meaning, and by extension, agency, becomes contested. For sport, this contest unfolds in strategic recalibrations and ethical tensions that will shape the next decade of athlete-fan engagement.

### Strategic reconfiguration of athlete branding

Athlete branding has always involved narrative construction, transforming lived experiences into marketable stories. What is novel in the era of augmented authenticity is that this storytelling is increasingly co-authored by generative systems. Athletes now deploy AI to scale their presence, maintain fan engagement across time zones, and respond to algorithmic cues with machine-generated precision. As Bleier et al. (17) describe in the context of the creator economy, this logic of co-production promises efficiency and reach, especially for athletes without the institutional machinery of elite sport behind them. This creates new pathways for strategic autonomy. Athletes can bypass traditional intermediaries, clubs, federations, media platforms, and engage directly with fans through synthetic yet emotionally resonant content. For some, especially from underrepresented groups or emerging economies, generative AI may serve as a levelling force, offering visibility and voice in a crowded digital field.

However, this autonomy is technologically conditional. Athletes now depend on the platform infrastructure and the platform's machine learning models and audience analytics that shape how their brand is perceived. As content is increasingly optimised for engagement metrics rather than personal meaning, the brand risks becoming algorithmically fine-tuned for virality but cosmetic in its personality. This mirrors Harari's fear that in outsourcing narrative construction to algorithms, humans risk becoming legible but shallow versions of themselves.

The strategic challenge becomes one of narrative sovereignty. How can athletes retain authorship when part of their identity is automated and potentially synthetic? In Scenario 2 (algorithmic co-production), this tension is managed. In Scenario 3 (synthetic substitution), it may collapse entirely. In such futures, the brand can function independently of the human who originated it, raising strategic concerns about what will be the athlete's legacy and post-career monetisation. The traditional brand principle of self-congruence (4), that branding is most effective when it reflects the athlete's true self, must now contend with hybrid authorship. Congruence is no longer just between self and message, but between self and system.

### Ethical tensions in hybrid identity production

With strategic power comes ethical complexity. As athlete branding shifts from expression to simulation, the moral status of authenticity, and what is being represented by who(m) must be re-examined. At the heart of this is the ambiguity of authorship and accountability. When AI generates athlete content, be it a motivational message, an apology, or an endorsement, who is responsible for the emotional impact or reputational fallout? Traditional legal frameworks grounded in intention and direct control are poorly equipped to adjudicate liability in hybrid content ecosystems. Xu and Baghaei (14) highlight the growing disjuncture between AI capability and governance readiness across sectors, with sport being particularly exposed due to its emotional salience, its irrational passion and consequently its commercial intensity. Audience deception is another concern. If fans are unaware that they are engaging with synthetic content, their emotional investment risks being built on false premises. This challenges the parasocial bond, a foundational concept in sport marketing (5, 7), which relies on the perception of genuine human connection. When that connection is simulated by algorithms, fans may feel betrayed upon discovering the illusion, or worse, become indifferent to the source altogether. This is the ethical hazard of emotional misrepresentation—the manipulation of affect without informed consent. The risk intensifies with what might be termed “performative algorithmicism,” the use of AI to craft highly optimised “authentic” content tailored to platform signals and audience sentiment. As Gao et al. (13) suggest, AI-generated emotionality may outperform human sincerity in terms of engagement. But at what cost? Athletes could find themselves pressured to maintain AI-enhanced versions of themselves, consistent, agreeable, and always “on brand.” The psychological toll of this hyper-managed identity, especially among younger athletes, remains understudied but likely significant. Lastly, temporal consent becomes critical. Once synthetic identities are created, they may persist beyond the athlete's control, used in future endorsements and extended into digital campaigns or virtual environments. In Scenario 3, these identities may operate independently, raising questions about posthumous rights and the commodification of the self over time. This calls for urgent institutional responses. A framework of ethical athlete branding in the AI era therefore could include clear origin tracking for content, distinguishing human-authored from machine-generated material; informed disclosure protocols so fans know when they are engaging with synthetic content; revocable licensing agreements that allow athletes to retract the use of their synthetic likeness as contexts evolve; and psychosocial safeguards to prevent emotional labour displacement and digital burnout. The strategic allure of augmented authenticity lies in its ability to amplify identity and democratise branding beyond institutional limits. But its ethical demands concern the very fabric of sport as a human-centred enterprise. If athletes are to remain more than avatars of engagement, if they are to retain their agency, dignity, and relational authenticity, then sport governance, fan culture, and platform design must evolve in tandem with the technologies that now co-author the story of sport.

## Governance implications of augmented authenticity

The integration of generative AI into athlete branding, what I have termed augmented authenticity, not only alters how identity is constructed but also demands a fundamental rethink of how identity is governed. The scenarios outlined in this paper illustrate that we are entering a transitional zone in which athletes are not just representing themselves but are being represented by machines, in forms that blend original self with algorithmic optimisation. This shift calls for governance frameworks that can both protect athlete autonomy and uphold the conditions for trust, credibility, and psychological safety in a sport ecosystem increasingly shaped by synthetic media. In all three scenarios, human-led authenticity with AI support, algorithmic co-production, and synthetic substitution, new governance questions emerge around authorship and representation. If AI systems generate content that misrepresents, offends, or distorts the athlete's identity, who is accountable? How are rights to likeness and voice managed when content is platform-dependent and potentially autonomous? And how can fans trust the relational cues they receive if they no longer know who, or what, is communicating?

### Repositioning sport governance

Sport governance has historically focused on regulating performance, competition, and commercial rights. But in an age where the athlete is increasingly a platform-native, AI-augmented brand governance must expand to include identity stewardship. This could include content integrity infrastructure, such as blockchain-based origin tools, to track the authorship and manipulation of digital materials; ethical oversight mechanisms embedded in athlete unions or federations to review and guide the use of synthetic content; revocable and time-bound licensing agreements that allow athletes to withdraw their digital likeness or synthetic personas from circulation; and disclosure standards requiring clarity on what content is human-authored, machine-generated, or co-produced. These are not merely technical additions, they signal a shift from governance as control to governance as co-creation, where athletes are not just regulated entities but co-designers of the systems that shape their digital futures.

### Human-centred digital representation

The governance implications extend beyond athletes and touch on a broader philosophical concern, and that is what kind of digital society are we building when authenticity is algorithmically assembled? The author of this paper acknowledges a normative orientation, a desire to preserve something essential about what it means to be human in the way we represent ourselves, not only in sport but in all spheres of mediated life. This perspective does not presume that the future will unfold in favour of human-led authorship. Indeed, as Harari (1, 2) argues, it is entirely possible that we will come to accept synthetic identities as emotionally effective and that they are produced commercially superior and culturally dominant. Yet precisely because this future is not inevitable, governance must be viewed not only as a response mechanism but as a directional tool. If scenarios help us imagine divergent futures, governance offers a way to influence which of those futures becomes reality. It enables stakeholders, athletes, fans, institutions, and platforms, to collectively choose the ethical coordinates of sport's digital transformation. This also implies a shift in governance literacy. Athletes must be supported not only in managing performance and partnerships, but in navigating the ontological terrain of digital selfhood. They need frameworks for understanding how AI can alter not just how they are perceived, but how they exist in the cultural imagination, how their identity is stored, remixed, monetised, and remembered. In that context it is important to reiterate that perceptions of authenticity are culturally variable. In some contexts, collectivist norms may encourage acceptance of digital proxies whereas in strong individualistic cultures there might be a heightened demand for direct human presence. This is why there is a need for cross-cultural research before governance principles can be generalised.

### Sport as a test case for society

Athlete branding in the era of augmented authenticity offers a preview of broader challenges to come. As more professions and personalities become partially or fully synthetic CEOs, influencers, educators, even politicians, the governance dilemmas faced in sport today will likely become societal norms tomorrow. Sport, in this view, is not an exception but a test case for developing institutional responses to the deep entanglement of identity, AI, and emotional labour. The task ahead is not to resist technological progress, nor to romanticise a pre-digital past, but to craft a human-centred architecture for digital identity. One that honours the complexity of the self, preserves conditions for trust, and ensures that machines amplify rather than replace the human voice. Whether or not this vision can prevail is uncertain. But if we recognise that futures are plural, we retain the agency to shape them. As alluded to earlier in this paper, in regard to possible governance interventions, actionable steps may include athlete digital literacy programs, disclosure requirements for synthetic content and revocable licensing agreement to safeguard narrative sovereignty.

## Concluding reflections and future research directions

This paper has introduced augmented authenticity as a conceptual lens for understanding how athlete branding is being reshaped by generative artificial intelligence. In doing so, it has sought to move beyond superficial debates about “real” vs. “fake” content and instead interrogate the deeper relational, ontological, and governance implications of identity co-produced by humans and machines. Through three illustrative future scenarios, human-led authenticity, algorithmic co-production, and synthetic substitution, I have explored the strategic opportunities, ethical dilemmas, and institutional blind spots now emerging at the intersection of sport and AI. These scenarios are not predictions but provocations. They are structured imaginations of possible worlds that invite scholars, practitioners, and policymakers to reflect on the values they wish to preserve, the risks they are willing to accept, and the systems they must design to steward athlete identity in an AI-driven sport business. While this paper acknowledges its normative bias, rooted in a humanist concern for self-determination and emotional integrity, it also recognises the inevitability of technological integration and the complexity of the trade-offs it entails. In this paper I also acknowledge several methodological limitations. First, as a conceptual analysis, it relies on extrapolation rather than empirical testing. The scenarios presented are intended as heuristic devices to probe possible futures, not as forecasts of what will occur. Second, the evidence base underpinning the scenarios draws heavily on English-language literature and Western sport contexts, which may inadvertently bias the framework toward cultural assumptions. Third, scenario planning as a method inevitably simplifies complex socio-technical dynamics into discrete trajectories, and in doing so risks overlooking hybrid or unexpected futures. These limitations highlight the need for empirical research, for example, experimental studies on fan perception, ethnographic work on athlete digital labour, and cross-cultural comparisons, to validate and refine the concept of augmented authenticity. Future studies should develop measurement tools and conceptual models that differentiate between types and degrees of human–machine co-production in athlete branding. This includes exploring how audiences perceive and evaluate authenticity under algorithmic mediation. Research to understand how fans respond emotionally to synthetic athlete content is also needed. Under what conditions does AI-generated content maintain, erode, or enhance fan loyalty and parasocial connection? Digital labour and athlete wellbeing requires research focus where the psychosocial impacts of managing hybrid identities must be explored, particularly for young or emerging athletes. This includes examining the emotional labour involved in maintaining AI-augmented personas and the risk of alienation from one's own brand. Governance frameworks and regulatory design require interdisciplinary research to prototype institutional and technological mechanisms that safeguard athlete rights, ensure transparency, and define ethical boundaries in the use of generative AI. Finally, in the global digital universe we still require a better understanding of cross-cultural and socio-technical fit. Given that athlete branding is embedded in cultural and economic geographies, and technological contexts, comparative research across location, sports, and platforms will be critical to understanding how augmented authenticity manifests differently around the world. In the end, the rise of AI in athlete branding presents sport with a generational choice—to passively adapt to a logic of efficiency and synthetic presence, or to actively design a future where technology amplifies, rather than erodes, the core values that make sport a meaningful expression of human identity. This paper represents a first step in articulating that choice, and in offering stakeholders the tools to influence its trajectory.
